# Evaluation parameters for evidence-based practices for people with autism spectrum disorder: a narrative review of group and single-subject design studies

**DOI:** 10.1186/s41155-022-00213-3

**Published:** 2022-07-20

**Authors:** Cássia Leal da Hora, Ana Carolina Sella

**Affiliations:** 1 Paradigma, Behavioral Science and Technology Center, Rua Bartira, 1294, Perdizes, São Paulo, SP CEP: 05011-001 Brazil; 2 Aprendizagem em Pauta, Rua Olindina Campos Teixeira, 172/601, Maceió, Alagoas CEP: 57036-690 Brazil

**Keywords:** Autism spectrum disorder, Evidence-based practices, Social validity

## Abstract

Recommendations for using evidence-based practices have become increasingly common in services for individuals diagnosed with autistic spectrum disorder (ASD). The aim of this study was to conduct a narrative literature review to identify differences and similarities in evidence-evaluation criteria for group and single-subject designs that empirically support interventions for people with ASD. Data sources used in this analysis were reports and articles elaborated by different clearinghouses (i.e., National Autism Center, National Professional Development Center, and the National Clearinghouse on Autism Evidence and Practice). The criteria for evaluating evidence, as defined by these documents, contained specific components or quality indicators for each type of study design. The different criteria for evaluating evidence and for classifying the interventions (once evidence was evaluated) were identified and described. This manuscript discusses the need for (a) expanding the analysis beyond the evidence identified by different researchers and organizations such as the clearinghouses, (b) proposing interventions that are based not only on scientific evidence but also on social validity — which is directed by client idiosyncrasies, and (c) attention to the fact that EBPs should not be seen as static information regarding interventions with empirical support: evidence-based practices are the result of constant analysis of the intervention implementation data added to professional training and client values and context. Some additional issues and the study limitations are also presented.

## Background

The diagnostic classification autism spectrum disorder (ASD) (American Psychiatric Association, 2013 [DSM-5]; World Health Organization, 2019 [CID-11]) is mostly attributed to people whose behavioral repertoire is characterized by deficits in social communication learning and by difficulties to exhibit behavior variability. Interventions that aim at strengthening behaviors that are in deficit and at weaking behaviors that might harm global functioning can help supersede common issues that might derive from the disorder.

According to the latest studies from the Center for Disease Control (CDC) (Maenner et al., 2020), the prevalence for ASD is increasing in some geographic areas from USA. Currently, ASD prevalence is 1 to 54 children. One consequence of this is that there has been an increase in (a) the search for treatments or interventions that aim at lessening the deficits commonly found in the disorder, (b) the search for professionals trained to offer these interventions, and (c) concerns regarding the quality and effectiveness of services (Steinbrenner et al. [NCAEP], 2020). In an effort to help consumers of these services, health and education professionals recommend the search for professionals that implement interventions based in scientific evidence (e.g., National Autism Center [NAC], 2015; Ontario Association for Behaviour Analysis [ONTABA], 2017; Wong et al. [NPDC], 2014).

The preoccupation regarding the need to use practices that have been shown its effectiveness scientifically has been set forth at least since the beginning of the 1970s, in different health professions. At that moment in time, Archie Cochrane (1972) called attention to the fact that health professionals in England were not basing their practice in scientific evidence. Cochrane emphasized the importance of existing publications that continually describe randomized clinical trial results (Cochrane, 1972). He also suggested that there should be an effort to transfer and apply such results in clinical practice (Greenhalgh, 2004).

Since then, a series of efforts has been made to develop studies that revised the scientific literature aiming at disseminating scientific evidence to be applied in clinical practice. This has led to the movement called evidence-based medicine (EBM) which, up to this day, is based on the assumption that medical conducts should be implemented using the best scientific evidence that is available at a given moment.

The EBM movement grew stronger with Sackett et al. (1996)’s work in which they proposed that clinical practices must be based in a rigorous decision-making and implementation process, using the best available practices for that specific patient, “integrating individual clinical expertise with the best available external clinical evidence from systematic research” (Sackett et al., 1996, p. 71).

Besides medicine, other fields have been broadening their interest in the evidence-based practices (EBPs) paradigm. Among these fields are nursing (Domenico & Ide, 2003), physical therapy (Filippin & Wagner, 2008), odontology (Richards, 2008), speech therapy (American Speech-Language-Hearing Association [ASHA], 2004), and psychology (Goodheart et al., 2006). The tendency to adopt this paradigm has also spread to other professional fields, including behavioral educational and psychosocial services for people with ASD (Ontario Association for Behaviour Analysis, 2017).

In the last few years, there has been a growing movement, within and outside of behavior analysis, for the production and assessment of scientific evidence regarding recommended interventions for individuals with ASD (e.g., McGrew et al., 2016). One of the main purposes of this movement is to provide support for professionals and consumers of services for ASD, helping them in the decision-making process regarding the best intervention to be used by the service provider.

One way to identify and evaluate the body of evidence produced by various independent studies that investigate the effectiveness of treatments or interventions is to perform systematic literature review studies (e.g., Sampaio et al., 2011; Seida et al., 2009). This process consists of reviewing the existing literature based on specific guidelines to include studies in the analysis. After selecting the studies that compose the sample (aka primary studies), a critical analysis of their methodology and results is conducted, based on a priori defined criteria (Ontario Association for Behaviour Analysis, 2017). Interventions that meet the criteria are regarded as evidence based.

Scientific studies are developed based on diverse methodologies and epistemological assumptions. Even within the same area of knowledge, not all research studies are the same. Therefore, it can be challenging to identify studies with methodological designs that guarantee both their internal and external validity as well as produce reliable results to base professional decisions within a field of application.

Such challenge has been addressed by different researchers, research groups, and organizations called clearinghouses. This organizations aim to be a central and reliable source of scientific evidence on interventions, practices, products, programs, and policies for different areas with socially relevant demands. Clearinghouses have the function of identifying, analyzing, evaluating, and synthesizing research and their findings, based on criteria that range from information about participants to their effects on target behaviors. In general, clearinghouses establish parameters to define what constitutes high-quality scientific research. Specifically in relation to ASD, the role of clearinghouses seems to have become increasingly relevant in several settings, especially in helping researchers and practitioners. In the past few years, at least three different clearinghouses have conducted systematic reviews and have published summary reports listing interventions that were effective in improving the behavior of individuals with ASD.

The purpose of this study is to discuss the need for (a) expanding the analysis beyond the evidence identified by different researchers and organizations such as the clearinghouses, (b) proposing interventions that are based not only on scientific evidence but also on social validity — which is oriented by client idiosyncrasies, and (c) attention to the fact that EBPs should not be seen as static information regarding interventions with empirical support; evidence-based practices are the result of constant analysis of the intervention implementation data added to professional training, client values, and context. Additional issues and the study’s limitations are presented in the conclusions. Below, in the main text, we describe the clearinghouses, as well as a summary of their review and evidence classification process. We organized their presentation in chronological order.

### Systematic literature reviews on EBPs for individuals with ASD

The National Autism Center (NAC) is a nonprofit organization, dedicated to disseminating evidence-based information about ASD. The clearinghouse was created in 2006 and is considered May Institute’s Center for the Promotion of Evidence-based Practice. In general, NAC proposes to offer comprehensive and reliable resources for families, professionals, and various members of the community interested in ASD-related services.

In 2009, NAC published the National Standards Project (NSP) — a synthesis report that describes phase 1 of its first systematic review with the aim of evaluating the body of evidence produced by experimental intervention research with individuals with ASD. They selected studies on focused and comprehensive interventions. Articles from peer-reviewed journals whose implementation environment was school, home, community, vocational, or clinical settings were included. Participants were children with ASD with no significant comorbid conditions. The reviewers identified 11 practices as “established treatments,” 22 as “emerging treatments” (with some positive evidence or studies with poor quality), and 5 that without enough evidence to guide the decision-making of professionals. Finally, the review did not identify any study that has proved ineffective or harmful to participants.

Importantly, NAC’s synthesis report (2009) presents its own definition of the term “treatment.” For them, the term could represent an intervention *class* or *strategy*. The panel of reviewers regarded an intervention class as a combination of strategies that bared the main common features. An intervention strategy, in turn, consisted of therapeutic techniques that can be implemented in isolation.

In 2010, a second review was published, which investigated the effectiveness of ASD interventions, authored by the  Odom et al. (2010); National Professional Development Center on Autism Spectrum Disorder [NPDC]. They aimed to identify, disseminate, and promote the use of evidence-based practices (EBPs) for individuals with ASD of various ages, including adulthood (up to 22 years old). The organization’s work was funded by the Office of Special Education Programs within the US Department of Education. The resources directed to students, professionals, and family members stemmed from the collaboration of three universities and several of their institutes (Frank Porter Graham Child Development Institute, University of North Carolina at Chapel Hill, MIND Institute, University of Wisconsin at Madison, and University of California-Davis).

The findings of NPDC’s first review (Odom et al., 2010) showed 24 focused intervention practices that met EPB criteria. Advancing in the mission of translating scientific evidence into practice, a collaboration was established between the NPDC team and the Ohio Center for Autism and Low Incidence Disorders (OCALI) to develop online training modules on each EPB intervention. These modules are currently known as Autism Focused Intervention Resources & Modules (AFIRM) and can be openly accessed upon registration in the platform (https://afirm.fpg.unc.edu/).

In 2014, the findings of a new systematic review conducted by NPDC were published in the synthesis report entitled *Evidence-Based Practices for Children, Youth, and Young Adults with ASD*. The process of reviewing and evaluating evidence aimed to update the previous one and followed the same methodological terms, although the period considered in primary studies was extended. In this report, three additional interventions reached the EBP criteria, totaling 27 interventions with positive effects for people with ASD.

Unlike NAC’s classification, neither NPDC’s synthesis documents mention the existence of emerging interventions, with no evidence of proven effectiveness or ineffectiveness. They present the additional classification “other practices with some support” without any reference of how many. They consist of behavioral packages or focused interventions with some positive effects (see Table 8).

Funding for NPDC’s and AFIRM’s studies ended in 2016. Since then, the Frank Porter Graham Child Development Institute has assigned the National Clearinghouse on Autism Evidence & Practice (NCAEP) to continue NPDC’s work.

In 2015, NAC published the findings of the second phase of their search and assessment process of EBPs conducted in 2009. They aimed to perform a data update and verify whether any emerging interventions met the criteria to be considered established. They covered a longer period in years and expanded the age range of participants with ASD who took part in the primary studies to include those over 22 years old. Finally, the reviewers proposed an important term change to refer to interventions. In phase 2, instead of “treatment” or “strategies,” they began using the term “evidence-based interventions” as a label for evidence-based practices to be adopted by professionals in the applied field. The authors warned that although this terminology may differ from other similar research studies, this distinction is important to elucidate the difference between an evidence-based intervention and the broader structure of professionals’ decision-making processes when providing evidence-based practice services (Sackett et al., 1996).

In NAC’s synthesis report (2015), 14 interventions for children and young adults with ASD were identified as EBPs. In addition, they described 18 practices as emerging and 13 with unestablished effects. For adults, they found one intervention that meets the EBP criteria, one identified as emerging, and four with unestablished effects.

In 2020, the National Clearinghouse Autism Evidence Practice (NCAEP) completed a new systematic review that followed those performed by NPDC (Odom et al., 2010; Wong et al., 2014). With nearly identical methodological standards as the two NPDC reviews and an extended analysis period, the 2020 review presented a set of 28 interventions that show evidence of positive effects on the behavioral repertoire of children and young adults with ASD. They were sorted into three categories: evidence-based practice, manualized interventions meeting criteria, and practices with some evidence. The novelty in relation to NPDC’s former review was the addition of the second category. Category definitions, as described by each clearinghouse, are presented on Table 8.

As described, several systematic literature reviews have been conducted over the past decade aiming to define evidence-based interventions for individuals with ASD. However, there is an ample debate on what constitutes EBP in the fields of knowledge production and intervention with ASD. According to Kasari and Smith (2016), although several literature reviews on EBPs for individuals with ASD have been published, each used different criteria for evaluating primary studies, thereby hindering the analysis of such evidence. In theory, studies with good methodological quality would lead to reliable conclusions on the effects of the intervention under investigation. However, variable parameters can either aid or hinder a reliable interpretation of the effectiveness of interventions.

One of the aims of this study was to identify differences and similarities between the evaluation parameters of studies on ASD interventions with group and single-subject designs in clearinghouses’ summary documents. To this end, we conducted a narrative review of the literature focusing on summary documents that described interventions with positive evidence among children and young adults with ASD.

Considering that different clearinghouses used different criteria for evaluating and classifying evidence, we raise discussion topics based on our findings to alert the reader about the need to look beyond appearances. We also discuss the importance of examining the criteria laid out in each document to classify a study as effective and, by extension, certain interventions as evidence based. On the following section, we present the data collected from the synthesis documents that supported our critical analysis.

## Data systematization

Table 1 shows the organizations that authored the systematic reviews, the year of publication, the title of their respective documents, and the types of intervention (focused or comprehensive) presented on the primary studies analyzed.

**Table 1:** Documents/authors, year of publication, document title, and types of intervention that were analyzed

Organization/authors	Year	Title	Types of intervention
National Autism Center (NAC)	2009	National Standards Report	Focused and comprehensive
National Professional Development Center (NPDC; Odom et al)	2010	Evidence-based practices for children, youth, and young adults with ASD	Focused
National Professional Development Center (NPDC; Wong et al.)	2014	Evidence-based practices for children, youth, and young adults with ASD	Focused
National Autism Center (NAC)	2015	Findings and conclusions: National Standards Project, phase 2	Focused and comprehensive
The National Clearinghouse on Autism Evidence & Practice (NCAEP; Steinbrenner et al)	2020	Evidence-based practices for children, youth, and young adults with autism	Focused

Although five reviews were conducted, only three organizations account for their authorship (NAC, NPDC, and NCAEP). In addition, it is important to note that NCAEP was assigned to replace and continue the work of NPDC, maintaining most of the criteria and modus operandi developed by the organization that preceded it. These results indicate that, to date, only the Frank Porter Graham Child Development Institute (FPG) (i.e., the NPDC and later the NCAEP) and the NAC have performed extensive analyses of intervention practices focused on children and young adults with ASD; only NAC analyzed comprehensive interventions.

We must consider the differences regarding the period covered by different systematic reviews and the total interventions labelled as effective by each one. Table 2 shows that the total range in years/months in each review by different clearinghouses ranged from 2 years and 5 months (NAC, 2015) to 51 years and 9 months (National Autism Center [NAC], 2009). However, the total time range that composes the sample of reviewed primary studies was combined in NAC’s phase 2 synthesis report (2015), resulting in an analysis of research published over a period of 54 years and 2 months in total. Similarly, NPDC’s second review (2014) synthesized results from publications for a total period of 21 years as it also incorporated studies from the previous review. Although NCAEP performed the systematic review with the shortest time range, its synthesis report also combined research from the two previous NPDC reviews, with a systematization of evidence produced over a total period of 26 years.

**Table 2:** Period, number of years and months, and total number of EBPs described in each review

Document	Period included in review	No. of years and months	Total number of years and months^**a**^	Total no. of EBPs
NAC (2009)	Jan 1957 to Sep 2009	51 y, 9 m	51 y, 9 m	11
NPDC (Odom et al., 2010)	Jan 1997 to Dec 2007	10 y	10 y	24
NPDC (Wong et al., 2014)	Jan 1990 to Dec 2011	11 y	21 y	27
NAC (2015)	Sep 2009 to Feb 2012	2 y, 5 m	54 y, 2 m	14
NCAEP (Steinbrenner et al., 2020)	Jan 2012 to Dec 2017	5 y	26 y	28

^a^ Sum of the time range covered in initial plus further reviews by each clearinghouse

Another information that can be observed in Table 2 concerns the number of interventions labelled as either evidence based (NPDC, 2010, 2014; NCAEP, 2020) or established (NAC, 2009; NAC, 2015). NAC’s most recent review (2015) reports half as many evidence-based interventions (14) when compared to those identified by NCAEP (2020) (28) even though NAC’s publication period is twice as long. These results indicate that different analysis and categorization criteria have been used by different clearinghouses.

Table 3 shows the designs used by the studies that composed the sample reviewed by each clearinghouse. In NPDC’s and NCAEP’s reviews, studies with group designs that included statistical analyses and studies with a single-subject design that failed to present their findings via graphs for visual inspection were excluded.

**Table 3:** Study designs found in each clearinghouse review

Types of study design	NAC (2009, 2015)	NPDC (2010, 2014)	NCAEP (2020)
**Group designs**	^**a**^		
Randomized controlled trial (RCT)		x	x
Sequential multiple assignment randomized trial (SMART)			x
Quasi-experimental design (QED)		x	x
Regression discontinuity designs (RDD)		x	x
**Single-subject designs**			
Withdrawal of treatment (ABAB)	x	x	x
Concurrent multiple baseline	x	x	x
Multiple probe	x	x	x
Alternating treatments	x	x	x
Changing criterion design	x	x	x

^a^In NAC’s 2009 and 2015 reports, the types of group designs that composed the revised sample were not specified

The data published by NAC failed to specify the types of group designs used by primary studies. Only NCAEP (2020) identified a study that used the SMART design, although it had already been accepted by both previous NPDC reviews.

Table 3 also shows that reviews included studies that used five types of single-subject designs: withdrawal of treatment (ABAB), concurrent multiple baseline, multiple probe, alternating treatments, and changing criterion design. NPDC’s and NCAEP’s reviews explicitly reported the inclusion of studies with combined single-subject designs, although this is not described in NAC’s reports.

Table 4 shows the total number of studies with group and single-subject design included in the different reviews. NAC’s (2009; 2015) and NPDC’s (2010) reviews do not specify the total number of studies that used the different types of designs. The available data shows that the number of primary studies with single-subject designs is greater both in the NPDC (2014) and NCAEP (2020) synthesis reports. Notably, in the most recent systematic reviews, the number of studies with single-subject designs in the sample practically doubled (from 408 to 806), whereas those with group design increased over fourfold (from 38 to 165). These results indicate that scientific evidence within the area of ASD continues to be predominantly produced by single-subject studies. However, there has been an increase in studies with group designs.

**Table 4:** Total number of studies with group and single-subject designs included in each review

Document	Studies with group designs	Studies with single-subject designs
NAC (2009, 2015)	Not reported	Not reported
NPDC (2010)	Not reported	Not reported
NPDC (2014)	38	408
NCAEP (2020)	165	806

**Fig. 1:**
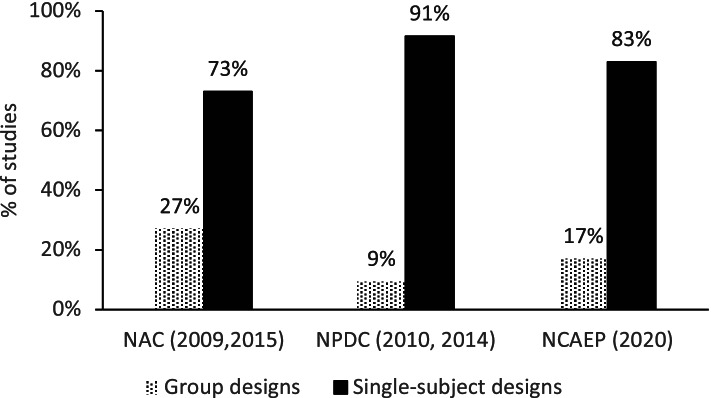
Distribution of single-subject and group design studies per clearinghouse

Although NAC’s (2009, 2015) and NPDC’s (2010, 2014) documents do not show the recurrence of each design in primary studies, NCAEP’s (2020) report presents the proportion of studies with group and single-subject designs in the four previous reports. These data are represented in Fig. 1 and corroborate the predominance of single-subject designs in the production of evidence within the area of ASD.

According to NCAEP’s (2020) report, multiple baseline is the most used among single-subject designs, followed by multiple probe, and a combination of designs (named others — for details, see Fig. 3.3 of NCAEP, 2020, p.32). Randomized clinical trials were the most used among group designs. Also, NCAEP’s (2020) report shows a considerable increase in the use of alternated treatments, multiple probe, and RCT designs when compared to NPDC’s (2014) report.

### Evaluation process of primary studies’ designs

To assess the experimental designs and their effects on primary studies, NAC developed their own assessment tools and strategies, which included three systems: (a) the Scientific Merit Rating Scale (SMRS), (b) the Intervention Effects Ratings, and (c) the Strength of Evidence Classification System.

The analysis established by the criteria of SMRS generated a score from 0 to 5 points for each primary study. The scale evaluated five key evidence components, namely research design, measurements of the dependent variable (DV), measurement of the independent variable (IV), participant ascertainment, and generalization and maintenance effects.

To assess the effect of interventions on participants’ target behaviors, NAC (2009; 2015) used the Intervention Effects Ratings. The effects produced by each intervention were sorted into four categories: beneficial (sufficient evidence to attest the effectiveness of the intervention), unknown (insufficient evidence to attest either the effectiveness or ineffectiveness of the intervention), ineffective (sufficient evidence to attest the ineffectiveness of the intervention), or adverse (sufficient evidence to regard the intervention as harmful).

The Strength of Evidence Classification System describes the quality, quantity, and consistency of the body of evidence directed towards ASD. This was the last evaluation system, and it sorted the effectiveness of interventions into “established,” “emerging,” and “unestablished.” Scores 3, 4, or 5 on the SMRS plus beneficial effects for the target behaviors signal sufficient scientific rigor by the study. Thus, it is possible to draw reliable conclusions about its effects and to infer that future studies would obtain similar results. Score 2 signals initial evidence of the effects of the intervention with beneficial effects on some target behaviors, thereby indicating a need for further, higher quality research. Scores 0 or 1 signals insufficient scientific rigor, making it impossible to label an intervention as beneficial, ineffective, or harmful.

Unlike NAC, to establish the review criteria for experimental designs and intervention effects, NPDC (2010, 2014) and NCAEP (2020) used analysis protocols explicitly derived from the existing literature. Among the references used to create the protocols were the studies by Nathan and Gorman (2007), Rogers and Vismara (2008), Horner et al. (2005), Gersten et al. (2005), and Chambless and Hollon (1998).

Based on the different types of research designs, different criteria were used to evaluate and score primary studies. Table 5 shows the criteria for rating the quality of evidence from studies with group designs. Table 6 shows the evaluation criteria for rating the quality of evidence from studies with single-subject designs.

**Table 5:**
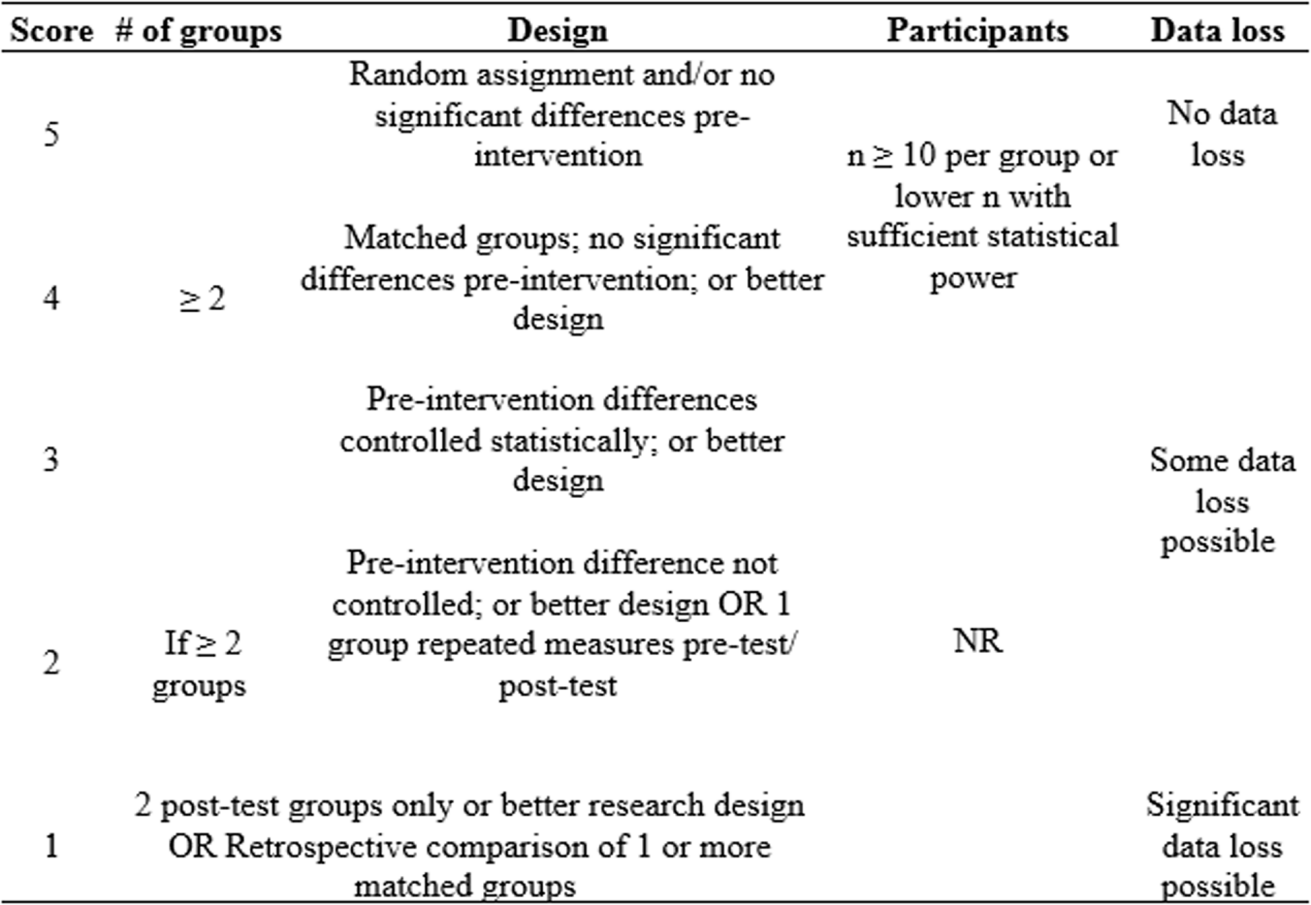
Evaluation criteria for group designs in the SMRS (adapted from NAC 2015, pp. 2428)

*NR* not reported

**Table 6:**
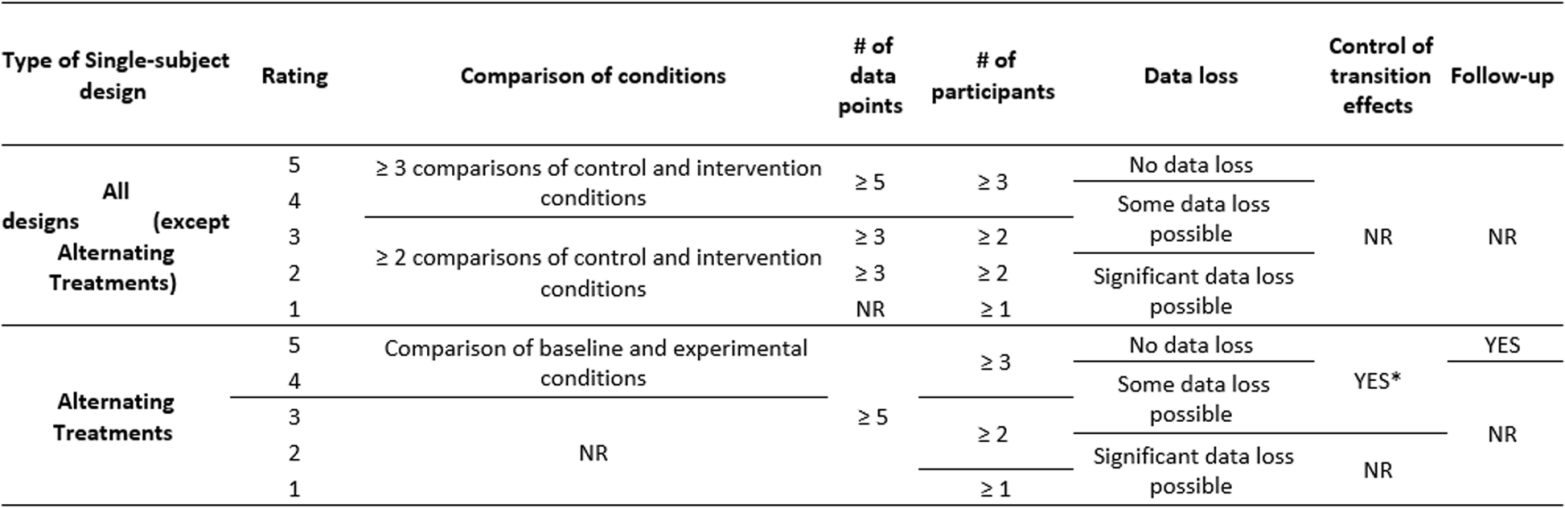
Evaluation criteria for single-subject designs in the SMRS scale (NAC 2009, 2015)

*SIG* significant, *NR* not reported; ^a^ transition effects were minimized by balancing key variables (e.g., time of day) or condition discrimination

The main methodological parameters evaluated are related to (i) the expected minimum number of groups per study, (ii) the way participants are organized among the groups, (iii) the minimum number of participants, and (iv) the tolerance regarding data loss. Higher scores are attributed to the studies with greater methodological rigor on the various key components of the scale. Also, maximum scores (5) are attributed to randomized group studies with no data loss. Some data loss is tolerated even in studies that receive a score 4. Lastly, the minimum number of participants is only specified for studies with scores 4 and 5 and not even reported for lower scores. These results indicate that attrition bias and the demand for a high total number of participants seem to be less relevant methodological parameters when it comes to investigations on interventions for individuals with ASD.

Table 6 shows that NAC’s main methodological parameters (2009; 2015) for evaluating single-subject designs are the number of conditions to which the effect of the intervention is compared, the minimum number of participants’ performance assessments (number of data points), number of participants, and data loss (as in the evaluation of group studies). Two additional parameters were the use of strategies to control for transition effects between conditions and a follow-up assessment. Alternating treatments and the other types of single-subject designs were evaluated separately. Table 6 also shows that higher SMRS scores are attributed to more rigorous evaluation parameters.

Table 7 shows the evidence quality indicators adopted by NPDC and NCAEP. The changes made by NCAEP (2020) to the evaluation form are highlighted in bold. Each row presents the 10 items used for evaluating studies with group designs (left part). The right part presents the 9 items for evaluating single-subject studies. In NPDC’s (2010, 2014) analysis, each item was classified dichotomously — yes or no answers. NCAEP’s (2020) review added a third “not reported” answer in its classification. For a primary study to be considered as evidence based, it had to meet all the items required for the type of design used. This criterion was adopted by the three reviews presented in Table 7 — NPDC (2010, 2014) and NCAEP (2020).

**Table 7:** Classification of evidence assessment items used by NPDC/NCAEP for studies with group and single-subject designs

NPDC (2010, 2014) NCAEP (2020)	
Group design quality indicators	Single-case design quality indicators
1. Does the study have experimental and control/comparative **(comparison)** groups?	1. Does the dependent variable align with the research question or purpose of the study?
2. Were appropriate procedures used to increase the likelihood that relevant characteristics of participants in the sample were comparable across conditions? **To meet this standard, one of the following criteria must be met: (a) participants were randomly assigned across study conditions, (b) participants were matched on key demographic variables, OR (c) researchers statistically controlled for effects of differing key variables to ensure equivalence of groups**	2. Was the dependent variable clearly defined such that another person could identify an occurrence or nonoccurrence of the response?
3. Was there evidence for adequate reliability of key outcome measures? And/or when relevant, was inter-observer reliability assessed and reported at an acceptable level?	3. Does the measurement system align with the dependent variable and produce a quantifiable index?
4. Were outcomes for capturing the intervention’s effect measured at appropriate times (at least pre- and posttest)?	4. Did a secondary observer collect data on the dependent variable for at least 20% of sessions across conditions? **Reviewers can select the** **not reported checkbox answer.**
5. Was the intervention described and specified clearly enough that *critical aspects could be understood?* **That it could be replicated by another interventionist?**	5. Was mean interobserver agreement (IOA) 80% or greater OR kappa of 0.60 or greater?
6. Was the control/comparison condition(s) described?	6. Is the independent variable described with enough information to allow for a clear understanding about the critical differences between the baseline and intervention conditions, or were references to other materials used if description does not allow for a clear understanding?
7. Were data analysis techniques appropriately linked to key research questions and hypotheses?	7. Was the baseline described in a manner that allows for a clear understanding of the differences between baseline and intervention conditions? **The reviewerscan select not reported for ATDs only. **
8. Was attrition NOT a significant threat to internal validity*?* **— ( ) Not reported checkbox answer**	8. Are the results displayed in graphical format showing repeated measures for a single case (e.g., behavior, participant, group) across time?
9. Does the research report statistically significant effects of the practice for individuals with ASD for at least one outcome variable?	9. Do the results demonstrate changes in the dependent variable when the independent variable is manipulated by the experimenter at three different points in time or across three-phase repetitions? **For ATD, there must be at least 4 repetitions of alternating sequence. For changing criterion, there should be baseline data plus three intervention phases.**
10. Were the measures of effect attributed to the intervention? (No obvious unaccounted confounding factors)	

Table 7 was adapted from the assessment forms on the quality of evidence presented in Appendix 2 of NPDC’s synthesis report (2014) and Appendix 1 of NCAEP’s report (2020). Boldface text refers to the contents present only in NCAEP’s (2020) version; *ATD*, alternating treatment design. Instructions for filling the form have been described only on NPDC’s (2014) report (“instructions: read each item and check the appropriate box. If you check “NO” at any time, the article will not be included as evidence for a practice”). In the NCAEP’s (2020) version, a checkbox with the answer “Not reported” was included in some items

Table 7 shows that the evaluation criteria used by NPDC and NCAEP are quite similar, focusing on the methodological parameters aimed at measures and arrangements that help in identifying the effects of IVs on the DVs. Unlike NAC’s evaluation system, there are no specifications as to the minimum number of participants, groups, number of DV measures, comparison conditions, or control of transition effects between the conditions. On the other hand, just as NAC’s evaluation, they also considered data collection reliability measures, attrition, and the separation of alternating treatment design’s evaluation criteria from the other designs.

Other important data contained in Table 7 concerns the description of what should be assessed by reviewers. We noted the use of terms that can hinder an objective review and lead to subjective interpretations of what each reader considers to be appropriate, for example, “appropriate” procedures, “sufficient clarity,” “adequately linked,” “main measures,” and “critical aspects” (items 2, 3, 4, 5, and 7 of the group design). In the evaluation of single-subject studies, terms such as “clarity,” “clearly,” or “sufficient” also appear (items 2, 6, and 7). This type of description — referred here as “not operational”— occurs both in group and single-subject designs. These results indicate that a reviewer or reader NPDC’s or NCAEP’s summary documents must have a prior understanding of what is “adequate,” “appropriate,” or “sufficient.” In the absence of operational definitions for such terms, they may rely on personal definitions.

Changes made to the assessment form on the latest systematic review (NCAEP, 2020), highlighted in bold in Table 7, add some information to descriptions that already existed in the previous assessment forms. Although such additions do not change the content or structure of the item being assessed, they seem to offer reviewers more specific descriptions. Given that NCAEP’s (2020) review proposes an update to NPDCs (2010, 2014), we can infer that the addition of information aimed at bettering the reviewers’ clarity of what must be evaluated and, eventually, the readers’ understanding of the criteria used.

Table 8 shows the categories used by each clearinghouse to classify the interventions, the definitions of each classification, and the criteria adopted by each organization to classify the different interventions used in primary studies. The number of categories created to classify the evidence and the effects of interventions ranges between two (NPDC) and three (NAC and NCAEP). None of the classification categories are identical, even when documents were authored by the same clearinghouse.

**Table 8:** Types and definitions of evidence classification and criterion used by each systematic review

Systematic reviews	Evidence classification/category	Definition	Criterion of empirical support
**NAC (**2009**,** 2015**)**	**Established**	Sufficient evidence is available to confidently determine that an intervention produces favorable outcomes for individuals on the autism spectrum. That is, these interventions are established as effective	(a) ≥ 2 GD or 4 SSD studies with ≥ 12 participants for which there are no conflicting results or at least 3 group design or 6 SSD studies with a minimum of 18 participants with no more than 10% of studies reporting conflicting results. (b) GD and SSD may be combined. (c) Peer-reviewed studies with SMRS scores of 3, 4, or 5 pts. (d) Beneficial intervention effects for a specific target. (e) These may be supplemented by studies with lower scores on the SMRS
	**Emerging**	Although one or more studies suggest that an intervention produces favorable outcomes for individuals with ASD, additional HQ studies must consistently show this outcome before we can draw firm conclusions about intervention effectiveness	(a) ≥ 2 GD studies or 2 SSD studies with ≥ 6 participants for which no more than 10% of studies reporting conflicting results. Conflicting results are reported when a better or equally controlled study that is assigned a score of ≥ 3 reports either (a) ineffective intervention effects or (b) adverse intervention effects; (b) GD and SSD may be combined. (c) Peer-reviewed studies with SMRS scores of 2 pts. (d) Beneficial intervention effects reported for one DV for a specific target. (e) May be supplemented by studies with lower SMRS’s scores
	**Unestablished**	There is little or no evidence to allow us to draw firm conclusions about intervention effectiveness with individuals with ASD. Additional research may show the intervention to be effective, ineffective, or harmful	(a) May or may not be based on research. (b) Beneficial intervention effects reported based on very poorly controlled studies (scores of 0 or 1 on the SMRS). (c) Claims based on testimonials, unverified clinical observations, opinions, or speculation. (d) Ineffective, unknown, or adverse intervention effects reported based on poorly controlled studies
**NPDC (**2010**,** 2014**)**	**Evidence based**	Specifies that an intervention is identified as EBPs if supported by the number of studies specified in the “criterion” column	(a) 2 HQ experimental or quasi-experimental design studies conducted by 2 different research groups, or (b) 5 HQ SSD studies conducted by 3 different research groups and involving a total of 20 participants across studies, or (c) there is a combination of research designs that must include at least 1 HQ experimental/quasi-experimental design, 3 HQ SSDs, and be conducted by more than one researcher or research group
	**Other practices with some support**	Some practices had empirical support from the research literature, but they were not identified as EBPs because it did not meet criteria established	Subdivided into (1) idiosyncratic behavioral intervention packages: behavioral packages not replicated across studies (i.e., combinations of EBPs and other practices to create interventions to address participant’s individual and unique goals) and (2) other practices with empirical support: focused intervention that there was an insufficient number of studies documenting efficacy, or there was a sufficient number of acceptable studies conducted by only one research group, or still, there were a sufficient number of SSD studies, but there were not a sufficient number of total participants across studies
**NCAEP (**2020**)**	**Evidence-based practice**	Interventions that have clear evidence of positive effects with children and youth people with ASD	(a) ≥ 2 HQ GD studies conducted by at least 2 different researchers or research groups, or (b) 5 HQ SSD studies conducted by 3 different investigators or research groups and having a total of at least 20 participants across studies, or (c) 1 HQ GD study and at least 3 HQ SSD studies conducted by at least 2 different investigators or research groups (across the group and single-case design studies)
	**Manualized Interventions meeting criteria**	Interventions that (a) are manualized, (b) have unique features that create an intervention identity, and (c) share common features with other practices grouped within the EBP classification	
	**Practices with some evidence**	Focused intervention practices, which did not yet have sufficient evidence to meet criteria for an EBP, but they had some empirical support	Not meeting criteria for EBP specially because there was an insufficient number of HQ studies providing support, few participants, or just one researcher or research group

*GD* group design, *SSD* single-subject design, *SMRS* Scientific Merit Rating Scale, *HQ* high-quality, *DV* dependent variable

Clearinghouses define their classification categories by demonstrating the positive effects of interventions on participants’ repertoires. Interventions must have sufficient scientific evidence to demonstrate either their effectiveness or ineffectiveness. Interventions with little to no evidence are called “other practices with some support” by NPDC (2010, 2014), “emerging” or “unestablished” by NAC (2009, 2015), and “practice with some evidence” by NCAEP (2020). Similarly, when interventions have sufficient evidence of effectiveness, clearinghouses adopt different classification categories (see Table 8).

Table 8 shows that the criteria adopted for evidence categorization also vary according to each clearinghouse. Although the three organizations base their analysis on the type of design (group and single subject), number of articles, methodological quality, number of participants, authorship of publication, the minimum number of primary studies, and the number of participants, the methodological quality required in the primary studies differs among clearinghouses. These findings indicate that assessing the body of evidence and classifying interventions may depend on the epistemological conception of each group regarding what should be considered as high-quality scientific evidence.

The data on evidence classification and its criteria must be highlighted because, although all reviews fundamentally aimed at identifying evidence-based interventions for individuals with ASD, their classification is based on different definitions and criteria. Such differences must be considered by practitioners, who will need to analyze these definitions and criteria in a more technical way to know what each category refers to.

Such a thorough analysis demands technical knowledge regarding the process of scientific production and its language which can constitute a barrier to its dissemination to consumers of services offered to individuals with ASD. On the other hand, a superficial analysis or the insensible consumption of information regarding EBP’s could generate misguided practices or, even worse, mislead the clinical practice in the field.

## Discussion

In recent years, summary reviews have been published to describe interventions that show evidence of positive effects in children and young people with ASD. Such publications can be considered a product of an expanding search for professional activities compatible with the EBP paradigm. The purpose of the present narrative review was to identify differences and similarities between parameters used to evaluate studies using group and single-subject design. Such parameters are described in clearinghouses summary reports regarding interventions for people with ASD.

Notably, science produces evidence in different ways, and different types of studies produce different levels of strength of evidence (e.g., Murad et al., 2016; Yates & Cochran, 1938/2009). According to Murad et al. (2016, p. 125), case reports are usually classified as studies that produce the least strong evidence, whereas randomized clinical trials produce evidence with greater strength. On the other hand, meta-analyses and systematic literature reviews are tools that have the function of “evaluating, synthesizing, and applying” the evidence produced by the studies. The clearinghouse documents analyzed here are systematic literature reviews and, therefore, serve as lenses that draw attention to certain interventions.

The results of the present study show that the three different organizations responsible for authoring five reviews analyzed publications from a combined period from 1957 to 2017. In chronological order, the results were published by NAC in 2009 and NPDC in 2010. The NPDC published its first update in 2014 and NAC in 2015. Finally, the NCAEP published and updated the work started by NPDC, authoring its first systematic review in 2020.

All organizations included research with group and single-subject designs in their sample of primary studies. The criteria for evaluating the evidence produced by such studies were defined by specific quality indicators for each type of design.

We found that NPDC and NCAEP used remarkably similar criteria which were based on the literature in the area. In addition, they incorporated criteria established by What Works Clearinghouse and the APA Division 12 Task Force. Furthermore, its conceptual structure is close to the one used by the Cochrane collaboration and other organizations, focused on patient/population/problem, intervention, comparison, and outcome (PICO https://linkeddata.cochrane.org/pico-ontology). The criteria used by NAC in both phases of their systematic review project were developed by the organization itself, and the report, despite citing classic studies (e.g., Sackett et al., 2000), does not cite the specific supporting literature for the design evaluation criteria.

In addition to the difference in the criteria for evaluating the designs and the effectiveness of the primary studies, we also found variability in the nomenclature used to classify the evidence of the interventions. Such variation, combined with varied classification criteria, can hinder the understanding of the information that bases the decision-making process of ASD-related professionals as well as stakeholders (e.g., family members and members of service funding agencies). The tables presented here can assist the analysis of evidence by people with little or no training in reading scientific articles.

At this point, we find it important to revisit the discussion on the function of systematic reviews when it comes to interventions for people with ASD. Several factors have influenced the emergence of the search for EBPs in this field among. One of these factors is the interests of different communities such as the scientific and professional communities and the stakeholders, including individuals with ASD. Systematic reviews should play an important role in the search for information that supports decision-making regarding interventions for this population. The results of such reviews should aid the translation of research findings into clinical practice (e.g., Cochrane, 1972; Green, 2008; Greenhalgh, 2004). However, as discussed by Green (2008), in addition to (a) an effort in translating arid, technical language into more palatable information and (b) disseminating such information to stakeholders, because the settings and conditions under which interventions are applied are complex (e.g., Dryden-Palmer et al., 2020), (c) measures such as social validity, external validity, attrition rate, and implementation reliability must be better described and discussed. Moreover, we advocate that the production of data by stakeholders when using EBPs must become more common and be somehow disseminated. As questioned by Green (2008, p. i20), “if it is an evidence-based practice, where is the practice-based evidence?”

Still from the perspective of the variations between clearinghouses classifications, there is a notable difference between the total of interventions that have reliably demonstrated positive effects for the target population in each case. For example, among the most recent reviews, 14 interventions were identified according to NAC’s criteria (2015) and 28 according to NCAEP. This difference is even more evident when considering the period covered by each review. NAC included articles from a period at least twice as long (54 years and 2 months) as the period reviewed by the NCAEP (26 years) and identified half as many interventions as effective. These findings indicate that the different criteria adopted by each clearinghouse impact the total number of interventions labelled as effective and allow us to raise the hypothesis that the criteria for evidence to be considered of high quality by NAC are stricter than NCAEP’s (former NPDC). Therefore, the different classification systems can lead to an intervention to be considered empirically supported by one organization rather than another. Ultimately, depending on the benchmark, stakeholders can make decisions based on evidence of effectiveness considered sufficient by one institution and insufficient by another.

Still regarding the transposition of evidence-based interventions to service provision, we must remember that, without implementation reliability (measure in which the treatment or intervention is implemented as described or planned), the effects obtained are likely to differ from those described in the literature (Brand et al., 2019). Data on implementation reliability and its effects on target behaviors are important for an evidence-based intervention to be effective in clinical practice.

For behavior analysts providing services to people with ASD, a warning is needed. Codes of ethics such as BACB’s (2014) and articles such Carr and Nosik’s (2017) and Weiss and Shook’s (2010) emphasize that, in addition to being based on scientific evidence, interventions must be based on the principles of applied behavior analysis (ABA). This implies that, even if a given intervention has evidence of effectiveness, it should not be implemented by behavior analysts if the principles of behavior analysis are not met.

Although a broad discussion about the point raised in this paragraph falls outside the scope of the present manuscript, we would like to emphasize that the use of EBPs by behavior analysts can be understood as an ethical duty for responsible professionals who work only within their training/experience limits and, therefore, consistently with the scientific principles that underpin our practice. However, as discussed earlier, aligning the provision of scientific evidence-based services with the client’s idiosyncrasies can be a challenging task. In the field of ASD, several pseudoscientific interventions as well as those with a body of emerging evidence are recurrently implemented by family members who seek to improve the life quality of a loved one. Behavior analysts may refuse to adopt practices that have no evidence and/or no support in behavior analysis. Interventions that are proven harmful (e.g., MMS (Miracle Mineral Solution) and chelation therapy), illegal (e.g., physical punishment for children in Brazil), or go against fundamental therapists’ values should not be accepted or adopted. Nevertheless, as discussed by Broadhead (2015), Rosenberg and Schwartz (2019), among others, behavior analysts must assess under what conditions they will refuse to work with cases in which nonbehavioral treatments are implemented and the effects that such refusal might have on the overall treatment plan and adherence.

We must remember that in the decision-making process of EBPs, choosing an intervention with empirical support is only one of the components that constitute the paradigm in question (e.g., McGrew et al., 2016). Even if a particular practice has not been included in a systematic review, the professional can assess the degree of reliability of the intervention based on both the number of published studies and its internal validity. In addition, professionals must choose the best available evidence based on its suitability to the specific demand of the client. As discussed by Spencer, Detrich, and Slocum (2012), it is important to strive for the use of the best available evidence based on the principle that little evidence, if well applied, is better than none. The use of single-subject experimental designs that help to identify the effects of different interventions on target behaviors (e.g., Mayer et al., 2019) can be a truly behavior-analytic conduct and committed to the social validity of the provision of services, insofar as it favors the distribution of control between the behavior analyst, “the specialist,” and the family/client through a joint and empirically validated decision-making process.

It is important to discuss that an evidence-based clinical practice goes beyond the identification of scientific evidence available to support interventions (e.g., McGrew et al., 2016; Slocum et al., 2014). Individual characteristics, culture, and the client’s preferences must always be considered (e.g., APA Presidential Task Force on Evidence-Based Practice, 2006). It is essential that technical and/or scientific knowledge is integrated into a broader decision-making process that will necessarily be affected by conceptual knowledge, practicum training, and the professional’s experiences and expertise (e.g., APA Presidential Task Force on Evidence-Based Practice, 2006). To avoid offering ABA-based services with incipient and possibly misleading analyses, it is essential that professionals improve their analytical skills (also understood as clinical expertise according to Leonardi, 2016) through supervised practice and through the development of critical reading and interpretation of scientific evidence (e.g., Mayer et al., 2019; Weiss, 2018). Without theoretical/conceptual consistency, it is very unlikely to achieve excellence in clinical practice, just as it is difficult to achieve effectiveness in the provision of services without considering their social validity for the people involved (e.g., APA Presidential Task Force on Evidence-Based Practice, 2006; Baer et al., 1987; McGrew et al., 2016; Slocum et al., 2014).

In addition, it is important that researchers, implementation professionals, and stakeholders are able to critically evaluate the criteria for analyzing evidence-based practices (e.g., McGrew et al., 2016). By having further information on both the process and the analysis criteria of each organization, people will be able to use information about the evidence more actively, deciding on those that are relevant to their practice and socially valid in the client’s context (e.g., Wolf, 1978). Without such understanding, which enables the critical consumption of information, meta-analyses and systematic reviews may be attributed the role of controlling entities that guarantee their influence based on the logic of authority-based practices (e.g., Gambrill, 1999; Gibbs & Gambrill, 2002). This can happen to the point of producing a dogmatic application of the reviewed interventions which bears a closer resemblance to “faith” rather than behaviors associated with a parsimonious, applied science that is committed to the social validity of services.

### Limitations

Due to the substantial amount of data presented in the synthesis documents and the endless possibilities of analysis, it should be noted that the topics discussed here were chosen under the authors’ bias. Further analyses and discussions can and should be performed from different viewpoints. To this limitation, we add the issue of an absence of specific and replicable criteria for choosing the data analyzed here. In addition, we note that the discussion and syntheses provided here are more qualitative than quantitative. Finally, it is important to highlight that both primary studies as well as literature reviews and meta-analyzes need to consider the social validity of the knowledge produced, as it only becomes relevant if the evidence is relevant to practice (e.g., Green, 2008).

## Data Availability

The datasets generated during and/or analyzed during the current study are available in different repositories, listed below. 1) National Autism Center [https://nationalautismcenter.org/national-standards-project/] 2) The National Professional Development Center on Autism Spectrum Disorder [https://autismpdc.fpg.unc.edu/sites/autismpdc.fpg.unc.edu/files/imce/documents/2014-EBP-Report.pdf] 3) The National Clearinghouse on Autism Evidence and Practice [https://ncaep.fpg.unc.edu/sites/ncaep.fpg.unc.edu/files/imce/documents/EBP%20Report%202020.pdf]
